# Supplementary material for: Neighborhood Violent Crime and Perceived Stress in Pregnancy
Source: Int J Environ Res Public Health. 2020 Aug 3;17(15):5585. doi: 10.3390/ijerph17155585 (PMC7432742; doi:10.3390/ijerph17155585)
Supplement: Supplementary file 1 [file ijerph-17-05585-s001.pdf]

Supplemental Table S1. Demographic characteristics of included and non-included participants from *Motherhood and Microbiome* cohort (n= 1, 943).

**Characteristic**	**Not included (n = 593)**	**Included (n = 1309)**	**P**
Age, mean (SD)	30.1 (5.7)	27.8 (5.9)	<0.0001
	n (col %)	n (col %)	
Nulliparous	249 (42.0)	580 (44.3)	0.34
Black	278 (46.9)	950 (72.6)	<0.0001
Married	302 (50.9)	963 (73.6)	<0.0001
Medicaid	215 (36.3)	792 (60.5)	<0.0001
PSS ≥ 30	63 (13.5)	281 (21.5)	0.0002
